# Complications with peripherally inserted central catheters (PICCs) used in hospitalized patients and outpatients: a prospective cohort study

**DOI:** 10.1186/s13756-016-0161-0

**Published:** 2017-01-28

**Authors:** Delphine Grau, Béatrice Clarivet, Anne Lotthé, Sébastien Bommart, Sylvie Parer

**Affiliations:** 1 Department of Infection Control and Prevention, CHU of Montpellier, 80 avenue Augustin Fliche, 34295 Montpellier Cédex 5, France; 2 UMR 5569 HydroSciences Montpellier, Team Pathogènes Hydriques Santé et Environnements, Unit of Bacteriology, Faculté de Pharmacie, Montpellier, France; 3 Clinical research and Epidemiology Unit, CHU of Montpellier, Montpellier, France; 4 Department of Radiology, CHU of Montpellier, Montpellier, France; 5 PhysMedExp INSERM U1046, UMR9214 CNRS, Montpellier, France

**Keywords:** PICC-related complications, Prospective follow-up, Inpatient and outpatient settings

## Abstract

**Background:**

Peripherally Inserted Central Catheters (PICCs) are widely used for hospitalized patients and among outpatients. Despite many advantages, PICC-related complications can occur such as infection, thrombosis or mechanical complications.

We aimed to evaluate rates and nature of PICC-related complications from insertion to removal and analyze risk factors of complications at baseline and during healthcare.

**Methods:**

We performed a prospective cohort study looking at PICC-related complication rates in the inpatient and outpatient settings of 163 patients over a 7-month period. Pertinent patient demographics as well as catheter-related factors were collected. The data were analyzed to identify catheter-related complications using univariate and multivariate analysis.

**Results:**

One hundred ninety-two PICCs were monitored for a total of 5218 PICC-days (3337 PICC-days for inpatients, 1881 PICC-days for outpatients). The overall complication rate was 30.2% (11.1 per 1000 PICC-days) with a mean time to onset of 16.1 days. Complications included occlusion (8.9%), accidental withdrawal (8.9%), infections (6.3%) including 9 local infections (4.7%) and 3 bloodstream infections (1.6%), venous thrombosis (1.6%) and hematoma (1%). Complication rate was higher in the hospitalization setting (36.1%; 14.38 per 1000 PICC-days) than in the outpatient setting (19.4%; 3.19 per 1000 PICC-days). Multivariate logistic regression analysis showed that the occurrence of occlusion was significantly associated with an age > 65 years (OR = 4.19; 95% CI [1.1–15.81]) and the presence of a pre-occlusive event the week before PICC removal (OR = 76.35; 95% CI [9.36–622.97]).

**Conclusions:**

PICCs appear safe in the inpatient and outpatient settings with low rates of infectious or thrombotic complications. Occlusion and accidental withdrawal were the most common complications, with age > 65 and catheter pre-occlusive event associated with an increased likelihood of catheter occlusion.

## Background

PICCs are widely used for patients requiring medium to long-term intravenous therapy in the inpatient and outpatient settings. As an alternative to central venous catheters (CVCs), PICCs allow for administration of medications requiring central venous access.

PICC-related complications include infection [1–3], thrombosis [4–6] and mechanical complications (i.e occlusion, accidental withdrawal) [7], with global rates of 15.9%, 34% and 40.7% respectively [8–10]. PICC-related bloodstream infections (BSI) rates of 2.1 per 1000 catheter-days in hospitalized patients and 1.0 per 1000 catheter-days in outpatient setting are reported [11]. Recent studies suggest that PICC-related BSI are less frequent than with other CVCs [12–14]. However, Chopra et al. showed that PICC-related BSI were as frequent as CVC-related BSI when infection rates were expressed by catheter-days [15]. Several factors could explain these diverging results, such as patient populations (oncology, pediatric patients) and therapies infused (parenteral nutrition, antibiotics). Moreover, the health-care setting could be a determinant factor in the occurrence of PICC-related complications [15, 16].

We performed a prospective cohort study of 163 patients in both the inpatient and outpatient settings over the period of 7 months to better clarify the impact of placement setting and patient co-morbidities on the incidence and nature of PICC-associated complications.

## Methods

### Study design: prospective cohort observational study

An unselected cohort was constituted by including every consecutive PICC inserted during a four month period (July through October 2010), regardless of demographic or medical status of the recipient patient or indication for PICC use. PICC placement was exclusively performed by the radiology department of the Montpellier University Hospital. Every PICC was prospectively and weekly followed until removal or until the end of the study in February 2011. Some patients were enrolled more than once, if they had more than one PICC during the inclusion period. All patients gave informed consent.

### Data collection

**Fig. 1:**
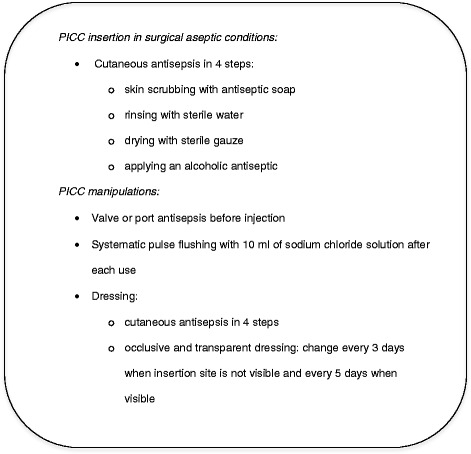
University Hospital of Montpellier recommendations for PICCs insertion and manipulations

At the time of PICC insertion, we collected data concerning patients’ demographic characteristics, comorbidities, immunosuppressive therapy, hospitalization ward and location of the patient 72 h after insertion. Data concerning the PICC were also collected: date of insertion, operator (junior or senior radiologist), treatment indication, rank of the PICC, device characteristics, compliance with pre-operative antisepsis protocol (Fig. 1), site of insertion, length and success of the procedure and type of PICC fixation.

Data were collected by performing patient chart review and/or phone calls to healthcare professionals involved in the patient’s care or directly to the patient. Information was obtained on patient outcome and occurrence of a catheter dysfunction or signs of infection. Data on PICC utilization concerned number of daily PICC accesses, frequency of dressings, type of antiseptic used for manipulating PICC lines, catheter flushing procedure and frequency of intravenous administration set change.

An information note about PICC care rules was given to the patient at time of placement and standardized protocols of antisepsis and PICC care were available in healthcare units of our hospital (Fig. 1). In instances of premature PICC removal, data were collected regarding the circumstances warranting the removal. If PICC-related infection was suspected, insertion site swabbing and catheter tip culture (according to Brun-Buisson method) were required, along with blood cultures if clinically relevant (general infectious symptoms). For each PICC, data were collected in a standardized questionnaire which was used throughout PICC follow-up.

The “inpatients” subgroup included all PICCs monitored from insertion to removal in a health-care setting and the “outpatients” subgroup included all PICCs monitored in the outpatient setting. PICCs used alternatively in hospital and outside were classified in a “mixed setting” subgroup.

### Definitions

Diagnoses of catheter related infections were established according to the French definitions:

- Confirmed catheter-related BSI was defined as the association of a positive blood culture in a patient having had a central line within 48 h prior to the onset of symptoms, AND one of the following criteria: 1) a positive culture of either catheter tip or exit site swabbing (≥10^3^ CFU/ml) involving the same organism as blood culture, 2) blood cultures from peripheral venous puncture and central lines positive with the same organism with a quantitative ratio (central sample/peripheral sample) > 5, or 3) a differential time to positivity > 2 h in favor of central line sample.

- Confirmed catheter-related local infection (LI) was defined as a positive culture of the PICC segment (≥10^3^ CFU/ml) with pus emerging from the exit site or a tunnel infection, with local manifestations of infection but no general signs of sepsis and negative blood cultures.

When all these criteria were not present or bacteriological culture not realized, or realized when the patient was under antibiotic therapy, we classified suspected infections as “possible infection”. When cultures remained negative (in the absence of antibiotics) or another cause of infection was diagnosed, the case was classified as “infection not confirmed”.

Local inflammation was defined by redness and/or soreness at the catheter exit site. General inflammatory signs were defined as isolated fever/chills without focal signs of infection.

Catheter-related venous thrombosis was defined by thrombus presence by ultrasonography.

Among catheter dysfunctions, pre occlusive events were defined as either a significant reduction of infusion flow or an impairment of blood back-flow. Lumen occlusion was defined by the permanent inability to flush the catheter or obtain blood back-flow.

### Statistical analysis

Quantitative variables were described as mean (+/-SD) or median (Q25-Q75) according to normality of distribution. For each variable, Odds Ratios (ORs) were obtained using logistic regression with the type of complication as the dependent variable. Each PICC insertion was considered as a new event. For each type of complication, a multivariate logistic model was then performed with all variables that were close to significance in the first model (*p* < 0.20). Otherwise, a *p*-value <0.05 was used for statistical significance. All statistical analyses were performed with SAS software (SAS Institute Inc, Cary, NC).

## Results

### Patients’ characteristics

From 12 July, to 21 October 2010, 194 PICCs were inserted in 163 patients with a median age of 61.7 years (range 14–96). Twenty-nine patients had more than one PICC inserted during this period: 27 patients had 2 PICCs and 2 patients had 3 PICCs. Demographic and medical characteristics of the population, as well as indications for PICC use are listed in Table 1.

**Table 1:** Descriptive characteristics of the patient population who had PICC inserted (*n* = 163)

Demographic characteristics	Value (%)
Age (years)	
< 25	7 (4.3)
25-40	15 (9.2)
40-65	68 (41.7)
> 65	73 (44.8)
Gender	
Male	94 (57.7)
Female	69 (42.3)
Comorbidities	
Diabetes	38 (23.3)
Solid tumor	23 (14.1)
Hematologic malignancy	37 (22.7)
Cystic fibrosis	4 (2.5)
AIDS	5 (3.1)
Immunosuppressive therapy	36 (22.1)
Hospitalization ward	
Oncology	34 (17.5)
Pneumology	29 (14.9)
Infectious diseases	25 (12.9)
Medicine	21 (10.8)
Geriatrics	15 (7.7)
Gastroenterology	13 (6.7)
Neurology	11 (5.7)
Endocrinology	10 (5.2)
Cardiology	10 (5.2)
Others wards	22 (11.3)
Other hospital	4 (2.1)
Indication for PICC placement*	
Antibiotic therapies	155 (79.9)
Hydration	98 (50.5)
Chemotherapy	53 (27.3)
Blood transfusions/samples	46 (23.7)
Total parenteral nutrition	44 (22.7)
Allo/autogeneic stem cell transplantation	19 (9.8)
Other medication	8 (4.1)

*Total may exceed 100% because many patients had more than one indication for parenteral treatment

**Fig. 2:**
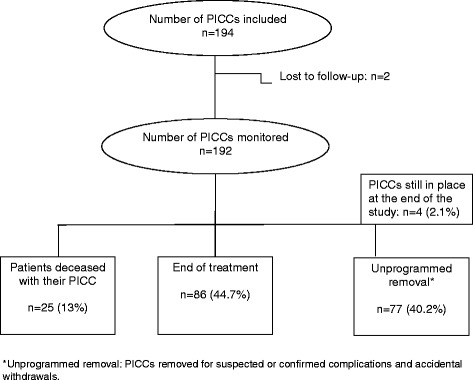
Study flow chart

Two PICCs were lost to follow-up, hence 192 PICCs were monitored from insertion to removal, for a total of 5218 PICC-days: 3337 PICC-days for inpatients (2700 in our hospital, 637 in other hospitals) and 1881 PICC-days for outpatients (Fig. 2). Overall mean PICC dwell time was 27.2 days (median 17 days; range 2–174), with mean dwell times of 23 days in the inpatient setting, 27.5 days in the outpatient setting and 46.9 days in patients managed in a mixed setting. Longest dwell times (>100 days) were observed mainly in oncology patients.

### Placement conditions and type of PICCs

All PICCs were inserted in the department of radiology, mostly by trained senior interventional radiologists (91.2%). Surgical aseptic conditions were reportedly applied in 100% of cases, in compliance with local recommendations. The PICC was inserted mostly for multiple indications (67%). The mean duration of the procedure was 15.20 min (3–120). No immediate complication was observed during or after insertion. A majority of PICCs were single lumen catheters (90.2%), medium size (96.9% were high flow 5 French devices), with a distal valve (79.4%) and mainly introduced under echographic control in the basilic vein (66.5%). Most PICCs were held in place with sutures (95.9%); 8 PICCs were attached with StatLock adhesive dressings (StatLock, Bard, Murray Hill, NJ, USA), which was privileged for young patients (4 had cystic fibrosis), with a mean PICC dwell time of 16 days, versus 27.7 days with suture.

### Modalities of PICC utilization

**Fig. 3:**
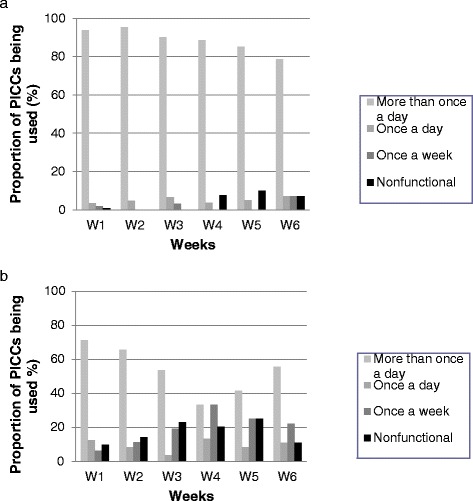
**a**: Frequencies of PICC utilization in Montpellier university hospital (inpatient setting group). **b**: Frequencies of PICC utilization in the outpatient setting

Frequency of PICC utilization (i.e: number of accesses per day) decreased over the course of care, with a higher proportion of seldom or never used PICCs in the outpatient settings (Fig. 3a and b).

Whatever the care setting, 55% of intravenous administration sets were replaced every 3–5 days; dressing frequency, type of antiseptic used and protocol of catheter flushing complied with local recommendations in 39.5% of the cases. Alcoholic chlorhexidine was mostly used in our hospital (78%), while povidone-iodine was mostly used in outpatient settings (94%).

### PICC outcomes

The global complication rate was 30.2% (11.1 per 1000 PICC-days) with a mean time to onset of 16.1 days. This rate was higher in the inpatient setting (36.1%; 14.38 per 1000 PICC-days) than in the outpatient setting (19.4%; 3.19 per 1000 PICC-days).

Main complications and outcomes of all monitored PICCs are shown in Table 2.

**Table 2:** Complications and outcomes of the PICCs (*n* = 192)

	General population (*n* = 192)	PICCs monitored in the inpatient setting (*n* = 133)	PICCs monitored in the outpatient setting (*n* = 31)	PICCs monitored in mixed health-care settings (*n* = 28)
Organic complications:				
Confirmed infections, value (%)	12 (6.3)	11 (8.3)	0	1 (3.6)
PICC-related BSI, value (%)	3 (1.6)	3 (2.3)	0	0
PICC-related LI, value (%)	9 (4.7)	8 (6)	0	1 (3.6)
Possible infections, value (%)	7 (3.6)	4 (3)	2 (6.5)	1 (3.6)
Infection not confirmed, value (%)	19 (9.9)	11 (8.3)	3 (9.7)	5 (17.8)
Deep vein thrombosis, value (%)	3 (1.6)	2 (1.5)	0	1 (3.6)
Hematoma, value (%)	2 (1)	1 (0.8)	1 (3.2)	0
Mechanic complications:				
Occlusions, value (%)	17 (8.9)	16 (12)	1 (3.2)	0
Accidental withdrawals, value (%)	17 (8.9)	14 (10.5)	2 (6.5)	1 (3.6)
Other causes of removal:				
End of treatment, value (%)	77 (40)	49 (36.8)	18 (57.9)	10 (35.7)
Other programmed removal, value (%)	9 (4.7)	5 (3.8)	2 (6.5)	2 (7.1)
Death, value (%)	25 (13)	20 (15)	2 (6.5)	3 (10.7)
PICC still in place at the end of the study, value (%)	4 (2.1)	0	0	4 (14.3)

Occlusions and accidental withdrawals occurred on average 16 days and 8 days respectively after PICC insertion, and the 3 episodes of deep vein thrombosis occurred 4, 5 and 39 days after PICC insertion.

### PICC-related infectious complications

Overall, 12 confirmed PICC-related infections (3 BSI and 9 LI) occurred, amounting to an infection rate of 2.3 per 1000 PICC-days. The overall PICC-related BSI and LI rates were 0.57 and 1.72 per 1000 PICC-days respectively.

All infections but one LI occurred in the inpatient setting: thus the global infection rates among in- and outpatients were 3.3 and 0.53 per 1000 PICC-days respectively.

The BSI occurred 6, 9 and 39 days after insertion; the mean time to onset for the 9 LI was 17 days. With respect to microbiology, 2 BSI were caused by coagulase-negative staphylococci and 1 by *Candida albicans*. Seven additional cases were possible infections according to definition: 4 inpatients presented a possible BSI, and 3 outpatients a possible LI. Nineteen PICCs were removed as a matter of principle in febrile patients, but infection was not ultimately confirmed.

### Other incidents

Forty-two pre-occlusive events occurred, with 55% of these occurring within the first week after insertion. Pre-occlusive events were managed by pulsed normal saline flush and/or heparin flush. Seventeen catheters (8.9%) were ultimately occluded, 16 of which had had a pre-occlusive event.

Twenty-five patients (15.3%) presented local or general inflammatory signs, with onsets occurring mostly within the first 4 weeks of PICC use. Interestingly, in 14 cases (56%), these precursor inflammatory signs were not followed by a positive diagnosis of catheter-related infection.

### Univariate and multivariate analysis

The univariate comparison of baseline patient characteristics between inpatient and outpatient subgroups showed significant differences concerning the number of cases of solid tumor (12 vs 29% respectively) and cystic fibrosis (0 vs 12.9%). Concerning infusion therapy, hydration was more frequent among inpatients (60.9 vs 16.1%) and so was total parenteral nutrition (27.1 vs 6.5%). The small number of outpatients did not allow to statistically compare subgroups in terms of outcome and risk factors.

Multivariate logistic regression analysis performed on the whole study population showed that the occurrence of PICC-related occlusion was significantly associated with 2 risk factors: age > 65 years (OR = 4.19; 95% CI [1.1–15.81]), and presence of a pre-occlusive event the week before PICC removal (OR = 76.35; 95% CI [9.36–622.97]). Interestingly, catheter dwell time was not associated with any of the complications.

## Discussion

Our single-center prospective study describes an unselected cohort of patients, among the first to benefit from PICCs in our hospital in 2010. As is still the case in our institution, PICC were placed exclusively by trained radiologists, and not by dedicated teams at patients’ bedside as in other countries. This specific procedure can limit the generalizability of our data.

Since then, to our knowledge, no prospective follow-up study has evaluated PICC complications among hospitalized and outpatients, regardless of the type of medication infused and patients’ conditions.

In the absence of published guidelines for healthcare professionals using PICCs at the time of this study, our hospital’s Infection Control and Interventional Radiology Departments had established local recommendations for PICC care, including a leaflet for home care. However, all the healthcare professionals were not yet familiar with these best practice rules, which could in part explain the high complication rate, although similar to rates reported in other studies [9, 17–19].

Guidelines for PICC care are now better defined and protocolized [20]. Recent studies suggest lower incidence rates of PICC-related complications, probably due to several technological novelties, better respect of the maximal sterile barrier precautions and improvement of compliance with evidence-based recommendations regarding catheter management in selected populations [8, 11, 21]. Bertoglio et al. documented 15% of complications leading to catheter removal in cancer patients but still concluded that PICCs represent safe devices for chemotherapy delivery, in particular during the first months after insertion [22]. In pediatric outpatients receiving parenteral antibiotic therapy, Kovacich et al. reported that 8% of PICCs required removal due to a complication (4.6 per 1000 catheter-days), underlining the need to discuss the relevance of PICC insertion and maintenance in children [23]. These studies underscore the importance of the type of patients, infused therapies and best practice recommendations.

In our study, incidence of lumen occlusions was high (8.9%), leading to catheter removal in all cases. Recent studies showed occlusion rates of 2.4% and 6% among hospitalized patients [19, 22] and 4.5% and 7.4% among outpatients [16, 24]. Our multivariate analysis identified age > 65 years and the presence of a pre-occlusive event as risk factors of lumen occlusion. In our study, catheter occlusion occurred on average 16 days after PICC placement, and was not associated with longer dwell times. We hypothesize that, because of the novelty of PICCs at the time of the study, healthcare professionals did not always follow instructions for the prevention of catheter obstruction and possibly did not heed the warning signs requiring timely prevention measures to be taken. French guidelines have since recommended systematic pulse flushing with saline after every use, heparin being used only as salvage therapy in some cases of lumen occlusion. Some published studies underline the role of nursing expertise in minimizing costs and complications and promote dedicated teams for safe PICC management [25, 26].

The second mechanical complication was accidental withdrawal of the catheter, which was as common as catheter occlusions (8.9%) and occurred mainly among hospitalized senior patients (mean age 70 years) with PICCs fixed on the skin by sutures. To prevent accidental removals, appropriate protection of the dressing is needful, particularly among elderly patients with behavioral disorders.

Concerning infections, we found a PICC-related BSI rate of 0.57 per 1000 PICC-days, which is lower than reported in the literature. For instance, Alenjo et al. reported an overall PICC-BSI rate of 3.13 per 1000 PICC-days in inpatients, higher in the ICU (4.79 per 1000 PICC-days) than in the non-ICU (2.78 per 1000 PICC-days) [27]; Chopra et al. also pointed out the ICU as risk factor for infectious complications with a PICC-BSI rate of 2.16 per 1000 PICC-days [28]. However, these differences can be in part explained by differing definitions of catheter-related BSIs between countries. Indeed, French studies that surveyed PICC-related complications among inpatients showed BSI rates comparable to ours [7, 9, 29, 30].

The prospective design of the study allowed us to register early local inflammatory signs in 25 PICCs (13% of the cohort), and to determine that less than half of these developed a confirmed infection. Moreover, 19 catheters were unnecessarily removed for suspected infections that were not confirmed. This underscores the need to apply rigorous diagnostic procedures for catheter-related infections (including differential blood cultures and insertion site swabbing), even if PICCs are seemingly easier to replace than CVCs.

We found a low incidence of symptomatic PICC-related venous thrombosis (1.6%; 0.57 per 1000 PICC-days). This result was similar to the incidence rate reported in the study of Kabsy et al. among oncologic patients (1.9%) but lower than in other published studies [5, 30, 31]. Turcotte et al. argued that, whereas the risk of infections related to CVCs and PICCs was similar, thrombotic complications were more frequent with PICCs and proposed a tailored approach in the choice of the most appropriate catheter [4].

We observed higher complications rates among hospitalized patients (14.38 per 1000 PICC-days) than in the outpatient settings (3.19 per 1000 PICC-days), with all the confirmed infections and 4/7 possible infections occurring in the inpatient settings. Smith et al. reported a 10-fold greater risk of PICC-BSI among hospitalized patients than outpatients and Chopra et al. demonstrated that PICCs were associated with a lower risk of infections (0.5%) that CVCs (2.1%) in outpatients [15, 32]. In our study, this can be explained by the differences between our in- and outpatient populations: the former had significantly more parenteral nutrition and daily catheter accesses, both known risk factors for catheter-related infections [11, 33]. Moreover, we might have underestimated the incidence of infectious complications, as PICC segments were not systematically cultured in the outpatient setting.

## Conclusion

In conclusion, this prospective study with a high-definition follow-up of every patient, allowed us to register precursor signs which were significantly related to later occurring complications such as lumen occlusion. PICCs appear safe to use in the outpatient setting, with acceptably low rates of infectious or thrombotic complications. Catheter occlusion and accidental withdrawal were the most common complications, both potentially avoidable with appropriate prevention measures.

## Abbreviations

BSI: Bloodstream infection
CVCs: Central venous catheters
LI: Local infection
ORs: Odds Ratios
PICCs: Peripherally inserted central catheters
